# A20 INTESTINAL SMOOTH MUSCLE AND FIBROSTENOSIS: TARGETING NR4A1 TO MODULATE PROLIFERATIVE SIGNALLING

**DOI:** 10.1093/jcag/gwab049.019

**Published:** 2022-02-21

**Authors:** J Lee, H E Szczepanski, K L Flannigan, S A Hirota

**Affiliations:** 1 Department of Physiology and Pharmacology, University of Calgary, Calgary, AB, Canada; 2 Physiology & Pharmacology, University of Calgary, Calgary, AB, Canada; 4 University of Calgary, Calgary, AB, Canada

## Abstract

**Background:**

Fibrostenotic Crohn’s Disease (CD), presenting with intestinal fibrosis and stricture formation, has a substantial impact on patient quality of life. Given our poor understanding of its etiology, we lack viable preventative and therapeutic methods. While much focus has been on the fibrotic component, recent studies have implicated the role of intestinal smooth muscle cell (SMC) hyperplasia/hypertrophy in stricture formation. These data suggest targeting SMC proliferation may provide benefit for fibrostenotic CD patients. NR4A1 (nuclear receptor subfamily 4 group A member 1) is an orphan nuclear receptor that has shown to regulate inflammation in experimental models of colitis and dampen SMC proliferation and fibrotic signalling in intestinal and non-intestinal systems. Thus, we sought to characterize the role of NR4A1 in regulating proliferative signalling in intestinal SMCs to determine whether it could be a therapeutic target for fibrostenotic CD.

**Aims:**

To determine how NR4A1 regulates intestinal SMC proliferative responses to mitogenic signalling.

**Methods:**

Primary intestinal SMCs were isolated from the colonic tissue of *Nr4a1*^*+/+*^ and *Nr4a1*^*-/*-^ mice. A commercially available human colonic SMC line was also used. EdU incorporation assays were used to quantify the relative proliferation of *Nr4a1*^*+/+*^ and *Nr4a1*^*-/*-^ SMCs in their basal or stimulated state (with platelet-derived growth factor (PDGF)-BB). In addition, NR4A1 was activated using selective agonists, cytosporone-B (Csn-B) and 6-mercaptopurine (6-MP). Differences in PDGF-BB-induced intracellular signalling was determined using western blotting of phosphorylated proteins after stimulation. Quantification of PDGF receptor transcript expression in Nr4a1^+/+^ and Nr4a1^-/-^ SMCs was done using qPCR. Finally, immunofluorescence was used to determine the localization of NR4A1 when stimulated with Csn-B, 6-MP, and/or PDGF-BB.

**Results:**

The proliferation assays showed that *Nr4a1*^*-/*-^ SMCs exhibit greater proliferation at baseline and when stimulated with PDGF-BB, compared to *Nr4a1*^*+/+*^ SMCs. However, this was not associated with any differences in the intracellular signalling directly downstream of PDGF receptor activation. Specifically, there were no differences in the intensity and temporal characteristics of Akt- and Erk1/2-phosphorylation between *Nr4a1*^*+/+*^ and *Nr4a1*^*-/*-^ SMCs. Interestingly, *Nr4a1*^*-/*-^ SMCs had less expression of *Pdgfrb*, the gene encoding PDGF receptor beta, when compared to *Nr4a1*^*+/+*^ SMCs. However, no changes in receptor expression were observed when SMCs were stimulated with Csn-B and 6-MP.

**Conclusions:**

We show that NR4A1 regulates basal and PDGF-BB-induced SMC proliferation, without directly altering the intracellular signalling cascades induced by receptor activation. Our data supports NR4A1 as a target to control the aberrant SMC proliferation that contributes to fibrostenosis.

**Funding Agencies:**

CIHR

